# Collapsing focal segmental glomerulosclerosis probably triggered by dengue virus infection - two case reports

**DOI:** 10.1590/2175-8239-JBN-2019-0237

**Published:** 2020-04-03

**Authors:** Patrícia Cruz Queiroz, Ana Elisa Souza Jorge, Plínio Henrique Vaz Mourão, Maria Goretti Moreira Guimarães Penido

**Affiliations:** 1 Santa Casa de Belo Horizonte, Serviço de Nefrologia, Belo Horizonte, MG, Brazil

**Keywords:** Glomerulosclerosis, Focal Segmental, Dengue Virus, Nephrotic Syndrome, Renal Insufficiency, Chronic, Glomerulosclerose Segmentar e Focal, Vírus da Dengue, Síndrome Nefrótica, Insuficiência Renal Crônica

## Abstract

The reported cases describe the association between collapsing focal segmental glomerulosclerosis (FSGS) and acute dengue virus infection. In both cases, patients were diagnosed with dengue virus infection and had a severe kidney disease, with nephrotic syndrome and acute kidney injury. Kidney biopsy was performed and showed collapsing FSGS.

The first patient, a 27-year-old man, was diagnosed with dengue virus infection and developed nephrotic syndrome after two weeks of illness. He was treated with methylprednisolone for three days and intravenous furosemide. This patient evolved well, although his renal function did not fully recover. The second patient, a 32-year-old man, was diagnosed with a milder clinical presentation of dengue virus infection. He had a past medical history of nephrotic syndrome in childhood, which might have caused its relapse. This patient was treated with intravenous furosemide and also did not fully recover renal function. These cases highlight the possible implication of dengue virus infection in the etiology of collapsing variant of FSGS. Healthcare professionals should be prepared to identify similar cases.

## Resumo

Os casos relatados descrevem a associação entre glomeruloesclerose segmentar e focal (GESF) colapsante e infecção aguda pelo vírus da dengue. Nas duas instâncias os pacientes foram diagnosticados com infecção pelo vírus da dengue e apresentaram doença renal grave, com síndrome nefrótica e insuficiência renal aguda. A biópsia renal revelou a presença de GESF colapsante.

O primeiro paciente, um homem de 27 anos, foi diagnosticado com infecção pelo vírus da dengue e desenvolveu síndrome nefrótica após duas semanas de doença. Ele foi tratado com metilprednisolona por três dias e furosemida endovenosa. O paciente evoluiu bem, embora sua função renal não tenha se recuperado plenamente. O segundo paciente, um homem de 32 anos, foi diagnosticado com apresentação clínica mais discreta de infecção pelo vírus da dengue. Ele tinha histórico de síndrome nefrótica na infância, o que pode ter causado a recidiva. O paciente foi tratado com furosemida endovenosa e também não recuperou plenamente sua função renal. Os dois casos destacam a possível implicação da infecção pelo vírus da dengue na etiologia da variante colapsante da GESF. Os profissionais de saúde devem estar preparados para identificar casos semelhantes.

## Introduction

Dengue virus (DENV) infection is a febrile disease caused by one of the four dengue viruses, which are serologically distinct. They belong to the Flavivirus genus and Flaviviridae family, and are called DENV-1, DENV-2, DENV-3, and DENV-4. The virus is transmitted by the *Aedes aegypti* mosquito vector. The infection may be asymptomatic or symptomatic. When symptomatic, it has a broad clinical spectrum, varying from mild to severe disease and even death. The disease is classified into three syndromes: dengue fever, dengue hemorrhagic fever, and dengue shock syndrome.^1^

Focal segmental glomerulosclerosis (FSGS) is a histologic pattern of injury characterized by the occlusion of a single or a group of glomerular capillary loops by sclerotic material and podocyte injury.^2^ It is divided into primary and secondary forms, which can be drug-induced, familial/genetic, and virus-associated forms, as well as those mediated by an adaptive response to a reduced nephron number. 

The collapsing variant of FSGS (FSGSc) is the most aggressive form^3^ and is often associated with human immunodeficiency virus infection^4^. However, it can be secondary to other virus infections, such as cytomegalovirus^5^, parvovirus B19^4^, hepatitis B and C^6^^,^ 7, and Epstein-Barr virus^8^. Other causes of FSGSc are pulmonary tuberculosis, leishmaniasis, autoimmune diseases (adult Still’s disease, lupus-like syndrome, mixed connective tissue disease), malignancies (multiple myeloma, hemophagocytic syndrome, acute monoblastic leukemia), and drug exposure (interferon alpha, beta or gamma, anabolic steroids, pamidronate, heroin).^5^

Considering the paucity of data regarding the link between FSGSc and dengue virus infection, we described two cases in order to illustrate this probable association.

This study was done according to ethics criteria and respecting the Declaration of Helsinki. Informed consent was obtained from both patients.

## Case Reports

### Case 1

A 27-year-old male presented to the emergency department with fever, myalgia, headache, retro-orbital pain, arthralgia, and asthenia. He had no known chronic ilness. Blood investigations revealed thrombocytopenia. He was diagnosed with classic dengue fever, without serology for diagnostic confirmation, and was treated with venous hydration and symptomatic medications. 

After two weeks of illness he developed progressive edema associated with foamy urine. He denied hematuria, dysuria, nocturia, or urine output reduction. Blood exams showed 8.0 mg/dL creatinine, 158 mg/dL urea, 5.8 mg/dL potassium, 2.7 mg/dL albumin, and significant dyslipidemia. Single urine sample showed protein 4+, rare granular cast, and protein/creatinine ratio (P/C) 7.1. The diagnosis of nephrotic syndrome (NS) was made. Screening results for HBV, HCV, CMV, EBV, and HIV were negative. The complements C3 and C4 were within the normal range. The anti-nuclear factor (ANA) was reagent 1:80 (fibrillar cytoplasmic pattern). 

The patient was treated with 1.0 gram methylprednisolone for 3 days, followed by prednisone 60 mg daily, and intravenous furosemide. There were clinical and laboratory improvement, with a 5-kg weight loss and decrease in serum creatinine to 4.6 mg/dL. He underwent an ultrasound-guided renal biopsy. 

**Figure 1:**
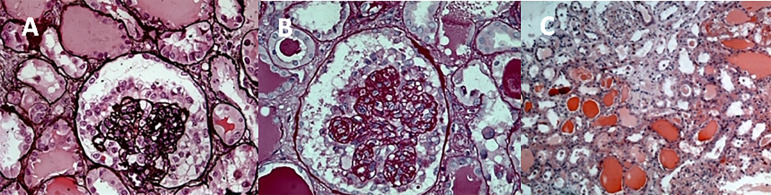
Bowman space dilatation with glomerular collapsed and podocyte hyperplasia. Several glomeruli showing collapsing glomerulopathy and tubular dilatation (A, B, C).

Light microscopy showed an increase in mesangial matrix with obliteration and frequent collapse of segments of glomerular capillary tufts, associated with fibrous capsular adhesions (synechiae) and marked podocytic hyperplasia in almost all 16 glomeruli sampled, constituting segmental sclerosis of collapsing pattern (Figure 1). The tubulo-interstitial space was enlarged by fibrosis (10-20%) with proportional tubular atrophy and discrete mononuclear inflammatory infiltrate. There were tubules irregularly dilated and filled with hyaline cylinders, determining a microcystic aspect. Immunofluorescence was positive for IgM and C3 with granular and irregular pattern (+++ / 3+), predominantly in mesangium. Other markers were negative. 

Polymerase chain reaction (PCR) for the detection of Flavivirus virus was positive for DENV infection in renal tissue. Unfortunately, the APOL1 genetic characterization was not performed. The patient evolved well, creatinine dropped to 2.0 mg/dL and the P/C decreased to 0.5. Currently, the patient uses only renin-angiotensin-aldosterone system inhibitors.

### Case 2

**Figure 2:**
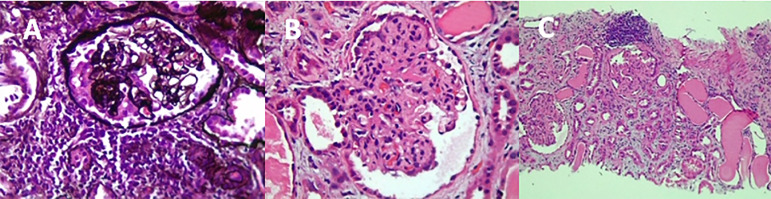
The mesangial matrix and the cellularity are slightly increased. The glomerular basement membrane exhibits foci of corrugation. The interstitial tubule space is enlarged by moderate fibrosis, tubular atrophy, and mononuclear inflammatory infiltrate (A, B, C).

A 32-year-old man was admitted to the hospital with a history of fever, myalgia, headache, and vomit for five days. His past medical history was significant for NS in childhood, hypothyroidism, and recent use of androgenic steroids. The physical examination revealed signs of hypovolemia. The patient received vigorous fluid resuscitation with isotonic saline. Five days later, he developed anasarca and noticed a reduction in urinary output. Initial laboratory tests confirmed diagnosis of DENV infection, via detection of viral antigen nonstructural protein 1 (NS1), and thrombocytopenia (36.000/mcL). Further tests revealed stage 2 of acute kidney injury (AKI) with elevated serum creatinine of 2.7 mg/dL from a baseline of 0.98 mg/dL, and hypoalbuminemia (1.4 mg/dL). Urine analysis showed proteinuria++ and a 24 h urinary protein excretion of 3.24 g. Screening for HBV, HCV, CMV, EBV, and HIV were negative. Immunological tests, including anti-neutrophil cytoplasmic autoantibodies (ANCA) and complements (C3 and C4), were negative or within the normal range. Due to the diagnosis of NS, a renal biopsy was performed. Light microscopy showed that six out of 13 glomerulus had segmental sclerosis with podocitary hyperplasia and collapse of the glomerular tuft. Tubular injury with cystic dilatation of the tubule was also noticed. Tubules were filled with hyaline casts (Figure 2). Immunofluorescence showed traces of IgG, C3, and kappa, with a granular segmental mesangial distribution. PCR for DENV infection was positive in renal tissue. APOL1 genetic characterization was not performed. The final diagnosis was FSGSc secondary to DENV infection. The patient evolved well after the same treatment prescribed for case 1. Creatinine dropped to 1.9 mg/dL and the P/C decreased to 0.7.

## Discussion

The classic DENV infection begins as an acute febrile infection, which coincides with a short period of viremia that ends on the third day after the onset of symptoms.^1^^,^ 9 Most clinical manifestations occur when viral levels are undetectable in peripheral blood. The DENV nucleic acid in peripheral blood mononuclear cells is also undetectable during the convalescence period.^9^ The types of kidney injury most often described in association with dengue infection are acute kidney injury and acute glomerulopathies, with large interstitial and tubular involvement. Data on chronic pathological findings of glomerular, epithelial, and podocyte damage as well as nephrotic proteinuria are scarce. 

Studies have suggested a relationship between APOL1 G1 and G2 genetic variants and kidney diseases,^10^ especially FSGS.^11^^,^ 12 APOL1 is one of the six members of the APOL gene family. It is the only secreted member of the family, and produced both systemically and locally in the kidney.^13^ The biological role of APOL1 in the podocyte remains unknown and the presence of renal risk alleles alone are not enough to induce kidney injury.^14^ In 2018, Araújo et al. described 13 patients with diagnosis of arboviruses (Dengue or Zika) and FSGS on kidney biopsy. The collapsing variant was identified in 11 patients and DENV was identified by PCR technique in seven of them.^15^ They proposed that dengue virus infection would act as a second hit to the development of kidney disease. However, a link between dengue virus infection and a patient’s genetic predisposition was not found. The authors suggested that further studies are necessary to investigate genetic risk factors in the Brazilian population.^15^ Previous reports in Brazil did not find an association between alleles risk variants of APOL1 and FSGSc.^16^

As previously mentioned, FSGSc is commonly associated with viral infection, most frequently with HIV and PVB19 infection.^3^ There are rare descriptions in the literature of this variant associated with arboviruses, such as Dengue virus.^15^ The mechanisms by which DENV may cause FSGS are not yet fully understood. It is known that the major pathogenic factor that induce lesions with a FSGS pattern is podocyte injury.^17^

A study with genetically modified mice expressing APOL1 G2 showed that mutated mice had a lower density of podocytes^17^ and this was a risk factor for glomerular diseases.^18^ Beckerman et al. demonstrated that renal disease, characterized by albuminuria, azotemia, glomerulosclerosis, and podocyte foot-process effacement, was caused by a podocyte-specific expression of renal risk variants.^18^ Experimental human models have showed that if the loss of podocyte density is greater than 40%, glomerular disease becomes progressive and irreversible, regardless of the cause, resulting in glomerulosclerosis.^19^ The expression of viral genes in kidney cells induced podocyte proliferation and dedifferentiation, apoptosis and fibrosis.^20^ In this context, we would suggest that the DENV enters into podocytes and initiates FSGSc.

The cases described herein presented with NS and loss of renal function. In case 1 the patient had a classic dengue infection (fever, myalgia, headache, retro-orbital pain, arthralgia, thrombocytopenia, and dehydration). He developed NS and AKI two weeks after the onset of the disease (creatinine 8.0 mg/dL; P/C 7.1). 

In case 2, the patient had a milder clinical presentation (fever, myalgia, headache, vomiting, and dehydration). He had a history of NS in childhood and dengue infection may have triggered its relapse. Both patients progressed with AKI and did not fully recover renal function. 

The case reports provide evidence that DENV might infect renal tissue and induce FSGSc. However, the pathophysiological mechanisms of arbovirus infections and their organ-specific complications have not been elucidated. Due to the severity of DENV infection, it is important that healthcare professionals identify similar cases and institute therapy in a timely manner. DENV infection should be considered an etiology of FSGSc in countries with a high prevalence of the disease. More studies are required to evaluate the role of DENV infection on FSGSc pathogenesis.
